# Cholangio-PET/CT with [^68^Ga]Ga-TEoS-DAZA for bile leak localisation after hepatobiliary surgery

**DOI:** 10.1007/s00259-026-08087-8

**Published:** 2026-07-20

**Authors:** Anke Werner, Robert Drescher, Felix Dondorf, Oliver Rohland, Utz Settmacher, Julia Greiser, Martin Freesmeyer

**Affiliations:** 1 Clinic of Nuclear Medicine, Jena University Hospital, Am Klinikum 1, 07747 Jena, Germany; 2 Clinic of General, Visceral and Vascular Surgery, Jena University Hospital, Am Klinikum 1, 07747 Jena, Germany; 3 Interdisciplinary Center for Clinical Research (IZKF), Jena University Hospital, Am Klinikum 1, 07747 Jena, Germany

**Keywords:** PET/CT, Hepatobiliary tracer, Biliary imaging, Bile duct injury, DAZA

## Abstract

**Purpose:**

To evaluate the clinical feasibility and potential utility of dynamic [^68^Ga]Ga-TEoS-DAZA PET/CT for the detection and localisation of bile leaks in patients after hepatobiliary surgery in a small, single-centre feasibility series.

**Methods:**

Ten patients with clinically suspected postoperative bile leakage and prior inconclusive imaging underwent [^68^Ga]Ga-TEoS-DAZA PET/CT between September 2024 and February 2026. Imaging was performed following intravenous administration of [^68^Ga]Ga-TEoS-DAZA (mean activity 214 ± 27 MBq). Dynamic PET acquisition was conducted for 60 min. Findings were analysed and classified according to the Nagano system where applicable, and their contribution to subsequent clinical management was assessed.

**Results:**

[^68^Ga]Ga-TEoS-DAZA PET/CT enabled localisation of bile leaks in 9 of 10 patients (90%). Leak sites were diverse including central and peripheral locations, as well as complex locations, such as ducts of Luschka and bilioenteric anastomoses. Mean detection time was 28.7 min post injection. PET/CT findings informed subsequent clinical management in all patients, including endoscopic, percutaneous, surgical interventions, or continued drainage management. No adverse effects were observed.

**Conclusion:**

Dynamic [^68^Ga]Ga-TEoS-DAZA PET/CT was feasible for bile leak localisation in this selected cohort, including complex and peripheral leak sites. Its physiological, antegrade imaging approach provided clinically useful information in patients with inconclusive or unsuccessful conventional imaging. Further prospective comparative studies are warranted to validate these findings and evaluate its diagnostic performance in larger cohorts.

## Introduction

Biliary leaks are serious iatrogenic complications following hepatobiliary surgery or liver-directed interventions, associated with substantial morbidity and mortality [1]. After cholecystectomy, bile duct injuries are severe complications linked to significant morbidity and mortality (up to 3.5%), requiring prompt diagnosis and treatment [2, 3]. After liver resection, the incidence of bile leaks has been reported to be even higher, ranging from 6.5% to 27.2% [4]. Their often nonspecific presentation may delay diagnosis, making early recognition critical for appropriate management [1]. Despite the availability of multiple imaging modalities, precise localisation of bile leaks remains challenging, particularly in complex postoperative settings. The novel radiotracer [^68^Ga]Ga-tri-ethoxysalicyl-1,4-diazepan-6-amine ([^68^Ga]Ga-TEoS-DAZA) enables functional hepatic positron emission tomography-computed tomography (PET/CT) imaging including antegrade depiction of the bile ducts due to its specific hepatic uptake and biliary excretion [5]. A recent toxicity study of the TEoS-DAZA precursor confirmed the pharmacological safety of the compound [6]. This analysis aimed to evaluate the feasibility of [^68^Ga]Ga-TEoS-DAZA PET/CT for bile leak localisation after hepatobiliary surgery and to assess its potential to improve therapeutic decision-making.

## Materials and methods

Ten consecutive patients with clinically suspected bile leakage, persistent postoperative biliary drainage after hepatobiliary surgery, and prior inconclusive, negative, or unsuccessful imaging including endoscopic retrograde cholangiopancreatography (ERCP), percutaneous transhepatic cholangiodrainage (PTCD) and magnetic resonance cholangiopancreatography (MRCP) were evaluated. Prior imaging was considered inconclusive if it demonstrated findings that were discordant with the clinical presentation and postoperative anatomy, precluding confident treatment planning. All patients provided written informed consent to undergo [^68^Ga]Ga-TEoS-DAZA PET/CT. The study was conducted in accordance with the Declarations of Helsinki and was approved by the institutional ethics committee (Reg. no. 2025-3873-BO-D).

Good manufacturing practice (GMP) compliant synthesis and quality control for clinical application were performed by labelling gallium-68 with the TEoS-DAZA precursor using an automated synthesizer (Scintomics GRP 3 V, Scintomics att, Fuerstenfeldbruck, Germany). The precursor (GMP grade) was obtained from Inflamed Pharma GmbH, Jena, Germany.

The mean administered activity of [^68^Ga]Ga-TEoS-DAZA was 214.0 ± 27.6 (median 215.5, range 152–256) MBq. No adverse or clinically detectable pharmacological effects were observed in any of the patients. Imaging was performed with patients positioned supine on a Biograph Vision PET scanner (Siemens Healthineers, Erlangen, Germany). A non-enhanced low-dose CT scan (120 kV, 50 mAs) of the upper abdomen was acquired for attenuation correction. Dynamic PET acquisitions of 20-minute segments were initiated in list mode simultaneously with intravenous bolus injection of the radiotracer (3 MBq/kg body weight, minimum 150 MBq) and continued for a minimum of 60 min. The PET data was reconstructed into 60-second frames. Images were reconstructed using the TrueX HD algorithm (Siemens Healthineers, Erlangen, Germany) with 3D attenuation-weighted OSEM (4 iterations, 12 subsets), followed by application of a 5-mm Gaussian filter, attenuation segmentation, and a 512 × 512 matrix. Images were analysed using syngo.via (Oncology module, Siemens Healthineers, Erlangen, Germany). Image interpretation was performed prospectively during routine clinical care. PET images were reviewed in consensus by nuclear medicine physicians and at least one hepatobiliary surgeon familiar with the respective patient’s surgical history and previous interventions. Prior imaging findings and knowledge of the postoperative anatomy were incorporated into the interpretation process to facilitate localisation of bile leaks in complex clinical scenarios. The Nagano classification was applied for descriptive purposes [7]. This classification was originally developed for post-hepatectomy bile leaks. Cases involving bilioenteric anastomoses, ducts of Luschka, percutaneous fistula tracts, or post-cholecystectomy leaks were classified in analogy to the Nagano system to facilitate comparison between cases. Subsequent management was reviewed retrospectively and discussed with the treating surgeons to assess the potential contribution of [^68^Ga]Ga-TEoS-DAZA PET/CT findings to clinical decision-making.

## Results

Between September 2024 and February 2026, 10 patients (6 female, 4 male) with a mean age of 63.2 years (range 42.3–76.3 years) were included in the analysis (Table 1). Patients with laboratory-confirmed evidence of postoperative bile leakage (persistent bilious discharge from an external drainage in nine, and from a fistula tract after removal of a drain in one patient) were referred for [^68^Ga]Ga-TEoS-DAZA PET/CT. The underlying clinical indications comprised a heterogeneous cohort of hepatobiliary and pancreatic pathologies, including intrahepatic and perihilar cholangiocarcinoma (*n* = 4), cholecystitis (*n* = 3), pancreatic cancer (*n* = 1), complex benign hepatobiliary conditions (*n* = 1), and renal cell carcinoma with liver metastasis (*n* = 1). All patients had undergone recent hepatobiliary surgery, most frequently partial liver resection (*n* = 6) or cholecystectomy alone (*n* = 3). Two patients underwent formation of bilioenteric anastomosis (BEA), and one patient underwent an associating liver partition with portal vein ligation for staged hepatectomy (ALPPS) procedure, respectively. The mean time interval between the most recent surgical procedure to [^68^Ga]Ga-TEoS-DAZA PET/CT was 29.0 days (range 3–85 days). At the time of the PET/CT examination, serum levels were 11.5 ± 6.4 (10.5, 3–27) µmol/l for bilirubin (reference range: <21 µmol/l), 32.2 ± 5.2 (32.5, 23–40) g/l for albumin (reference range: 35–52 g/l) and 138.5 ± 156.3 (93.5, 55–604) µmol/l for creatinine (reference range: 44–80 µmol/l for women; 62–106 µmol/l for men). As detailed in Table 1, prior imaging either could not be performed because of altered postoperative anatomy, did not demonstrate a bile leak despite persistent clinical suspicion, or yielded findings that were considered inconsistent with the patient’s postoperative anatomy and clinical course, thereby limiting confidence in subsequent treatment planning.

**Table 1:** Patient characteristics and imaging before [^68^Ga]Ga-TEoS-DAZA PET/CT

Patient no.	Age (years), sex	Primary Diagnosis / Procedure	Most recent surgical procedure	Prior imaging findings	Prior imaging summary
1	42.3, f	perforated cholecystitis with cholecystic-colonic fistula	open CCE, fistula repair	MRCP/ERCP: no definite leak location	negative
2	70.9, f	iCC	ALPPS procedure (right trisectorectomy)	ERCP: possible leak at resection surface	inconclusive
3 (Fig. 1)	68.5, m	pCC	left hemihepatectomy, BEA application	bile duct tube cholangiography: possible peripheral leak	inconclusive
4 (Fig. 2)	65.0, m	acute cholecystitis and gall bladder hydrops	open CCE	PTCD: failed, ERCP: not possible after Billroth II surgery	failed intervention
5 (Fig. 4)	63.7, f	recurrent liver cysts, CBD laceration during fenestration surgery	BEA application	bile duct tube cholangiography: no leak detected, ERCP: not possible due to BEA	negative
6	76.3, f	chronic cholecystitis with cholecystolithiasis	laparoscopic CCE	ERCP: no definite leak location	negative
7	49.3, f	pancreatic cancer, pancreaticoduodenectomy	atypical left liver-resection, excision of bilio-cutaneous fistula	PTCD: failed, ERCP: not possible after pancreaticoduodenectomy	failed intervention
8	59.0, f	iCC	left hemihepatectomy	ERCP: possible leak at liver hilus	inconclusive
9	70.4, m	iCC	extended left hemihepatectomy, BEA application	bile duct tube cholangiography: no leak detected	negative
10 (Fig. 3)	66.6, m	RCC with liver metastasis	resection of segment VII	ongoing external biliary drainage after ERCP/stent and surgical bilioma removal	inconclusive

ALPPS: associating liver partition and portal vein ligation; BEA: bilioenteric anastomosis; CBD: common bile duct; CCE: cholecystectomy; iCC/pCC: intrahepatic/perihilar cholangiocarcinoma; ERCP: endoscopic retrograde cholangiopancreatography; MRCP: magnetic resonance cholangiopancreatography; PTCD: percutaneous transhepatic cholangiodrainage; RCC: renal cell carcinoma

Dynamic PET/CT acquisition was performed over 60 min (three 20-minute list-mode acquisitions) in nine patients. In one patient (no. 10), the scan was discontinued after 20 min due to severe pain; however, bile leak detection was already feasible at that time. In two patients, additional acquisitions were performed (no. 2, 60–80 min; no. 6, 60–80 min, followed by a scan at 90 min after patient repositioning).

**Fig. 1:**
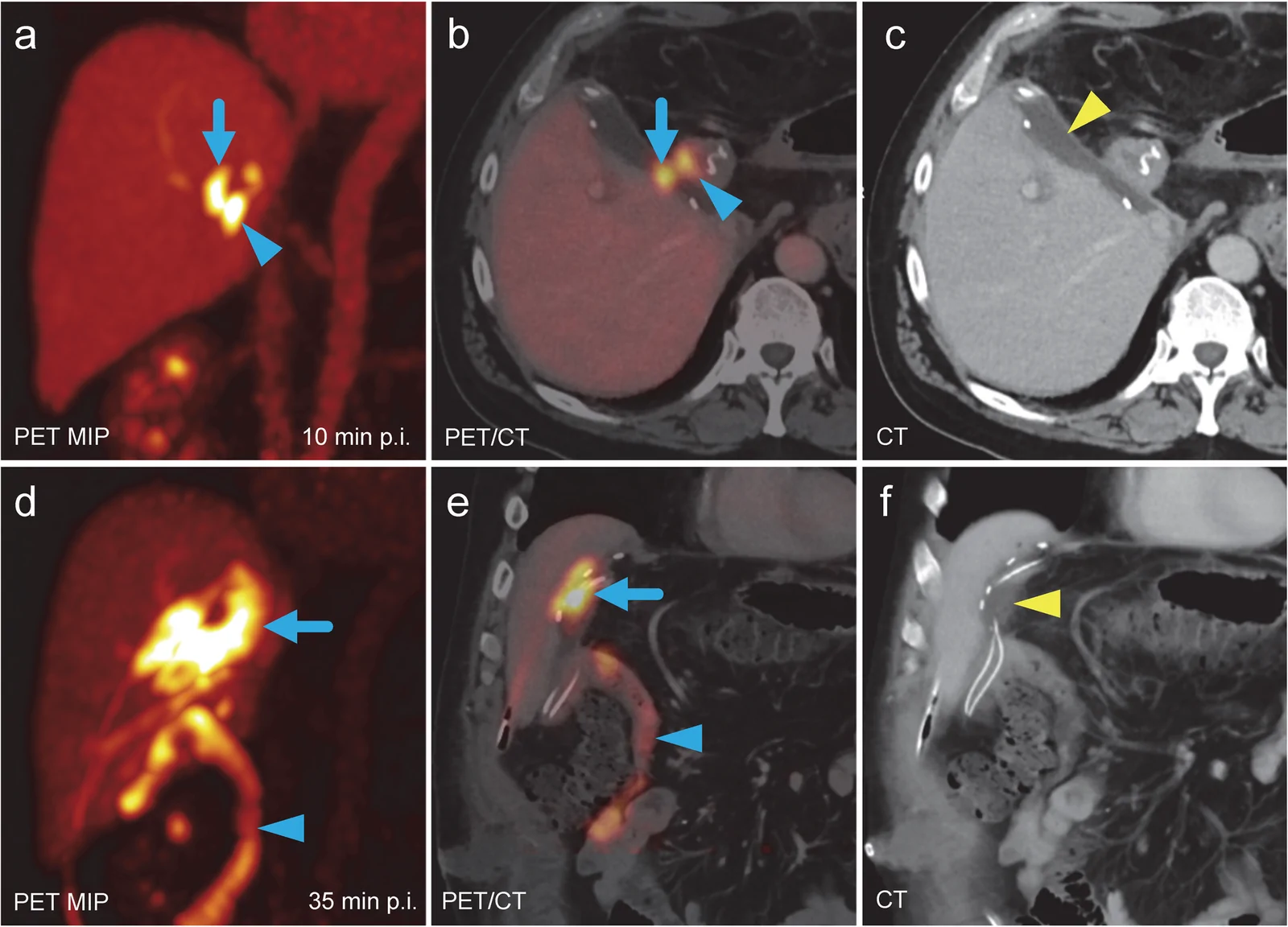
68-year-old man with perihilar cholangiocarcinoma (patient no. 3). Ongoing biliary drainage after a left hemihepatectomy with BEA application. Concurrent with bile crossing the BEA (**a/b**; arrowheads), a bile leak (**a/b**, arrows) into a perihepatic bilioma (**c/d**, arrowheads) was evident on PET/CT. Later, the bilioma fills further (**d/e**, arrows), with some bile transported in the small bowel (**d/e**, arrowheads)

**Fig. 2:**
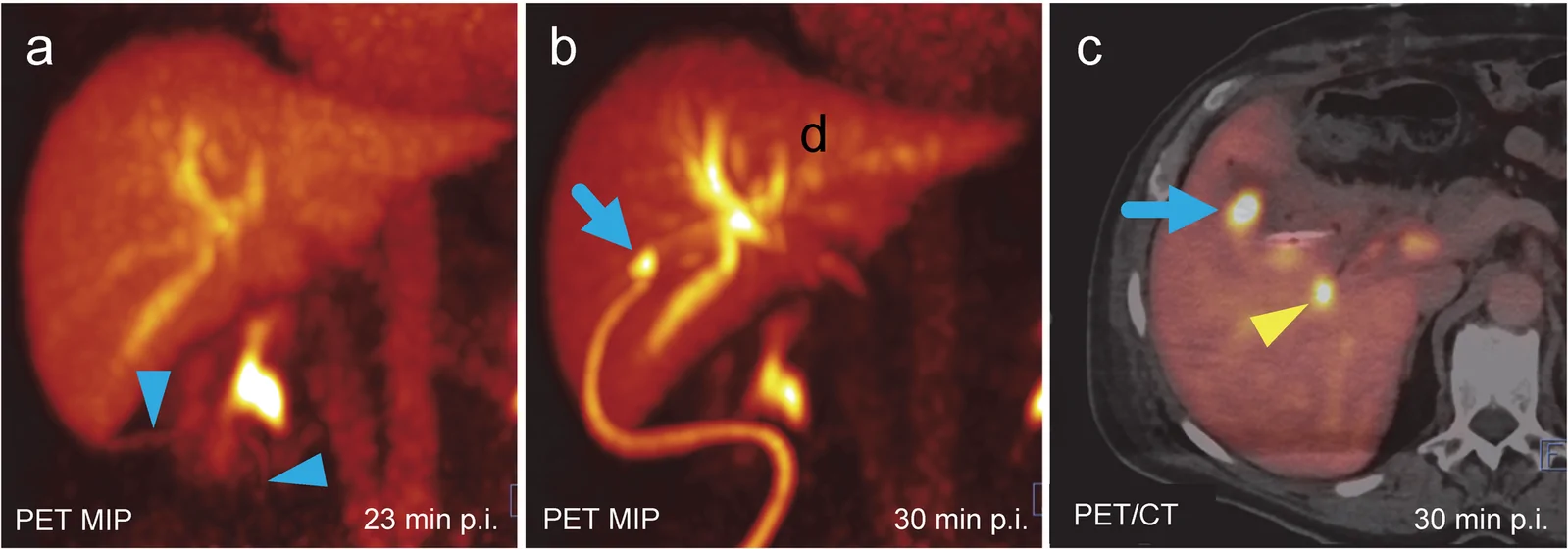
65-year-old man with persisting biliary drainage after conversion cholecystectomy (patient no. 4). On PET/CT, labelled bile began to appear in the subhepatic drainage 23 min after tracer injection (**a**, arrowheads). From 25 min, the leak site in the gallbladder bed close to the drainage became evident (**b/c**, arrows), consistent with an opened duct of Luschka. The second intense spot (**c**, arrowhead) is a central bile duct

**Fig. 3:**
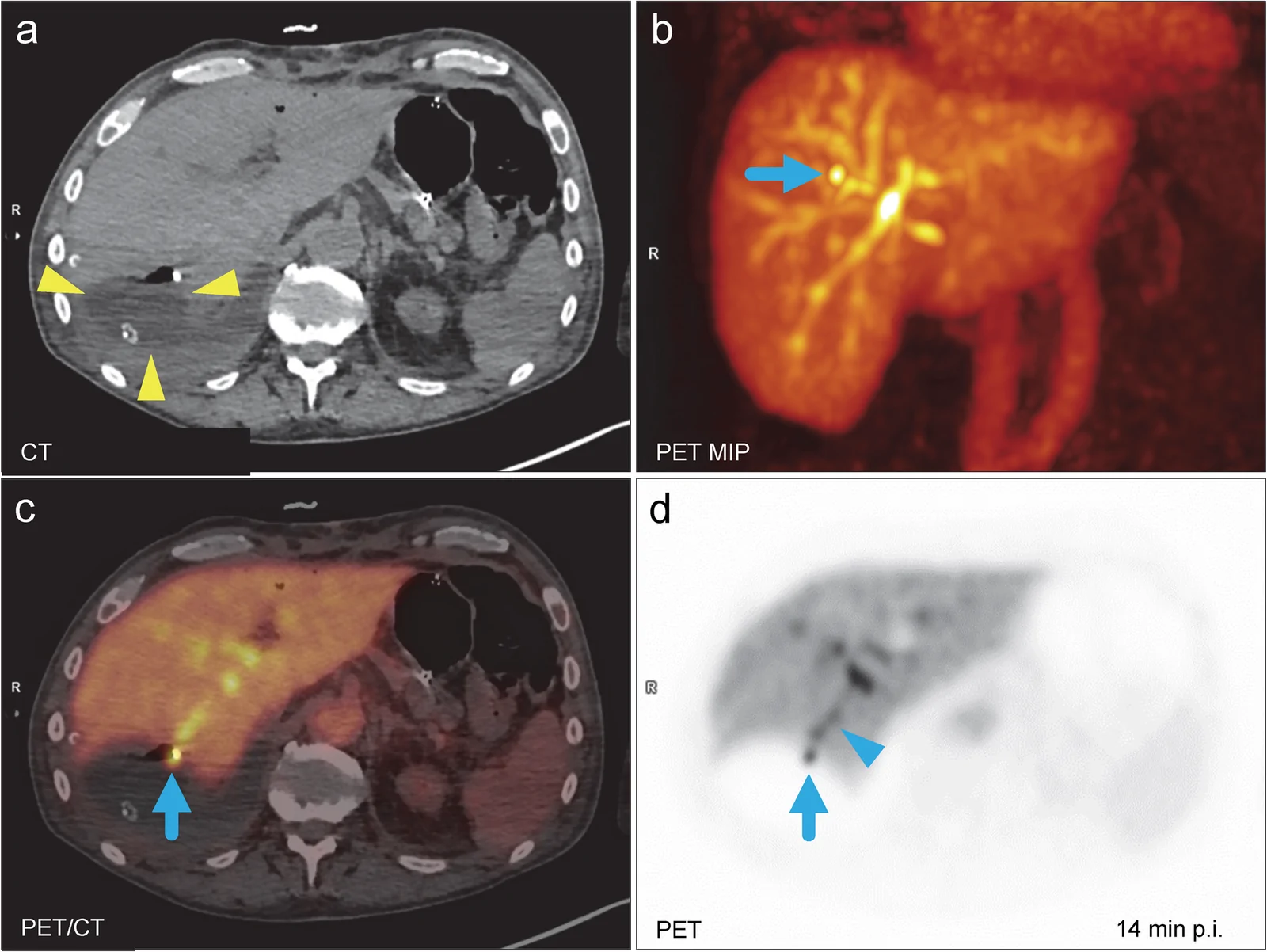
66-year-old man with renal cell carcinoma and hepatic metastasis (patient no. 10). CT shows a fluid collection after liver segment VII resection (**a**; arrowheads). On PET/CT, a bile leak is detected at the resection surface (**b-d**; blue arrows). The bile duct leading to the leak can be identified (**d**; arrowhead)

**Fig. 4:**
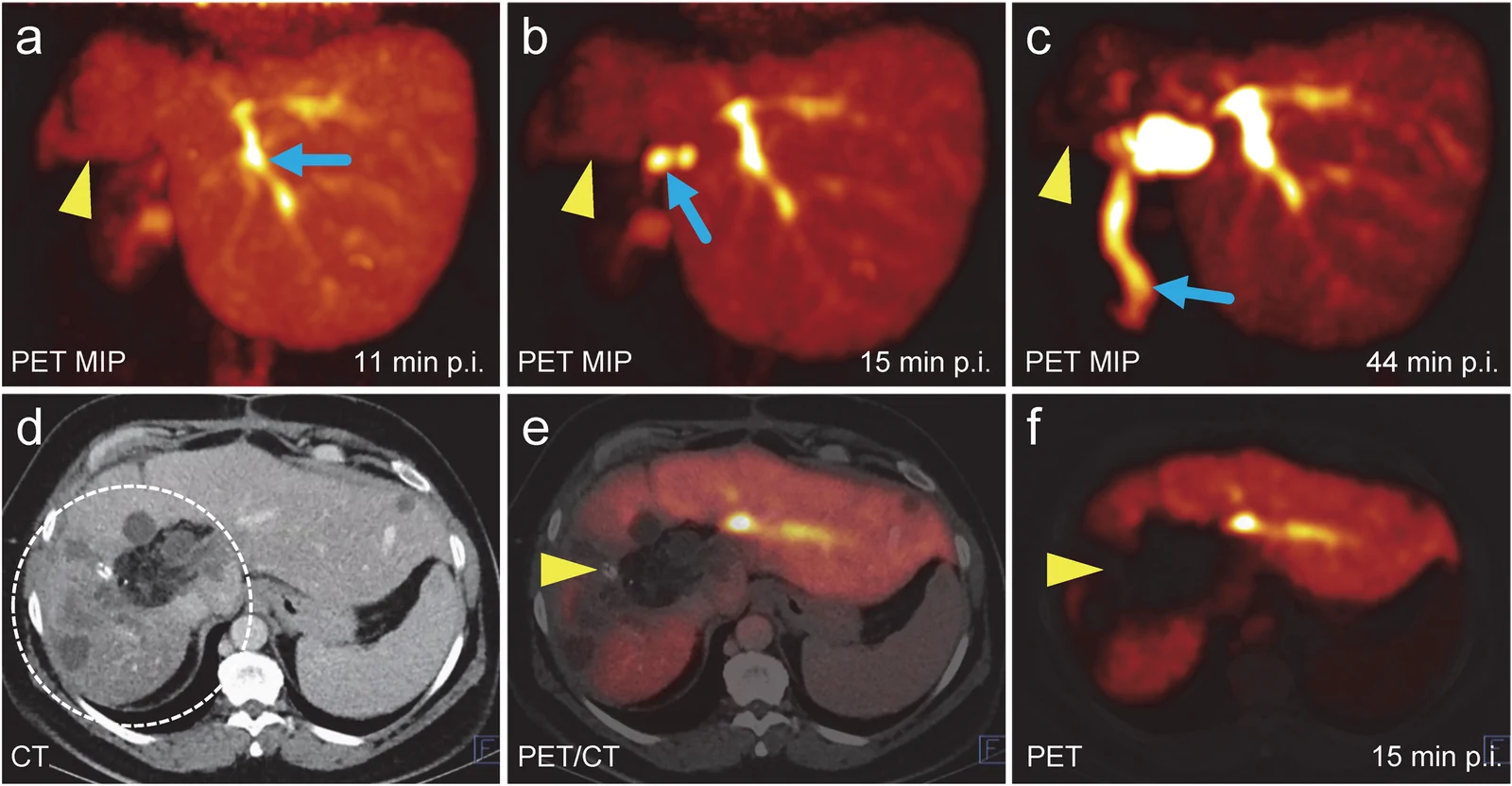
63-year-old woman (patient no. 5). No bile leak could be identified. Normal dynamic PET appearance of the hypertrophic left lobe with bile ducts appearing 11 min after tracer injection (**a**, arrow), bile crossing the BEA (**b**, arrow), and flowing into the small bowel (**c**, arrow). Uptake in the right liver lobe was low throughout (**a-c**, arrowheads) due to extensive tissue damage due to cysts and repeated surgery (**d**, circle). The drainage was located adjacent to the right lobe (**e**, arrowhead), in the region where no discernible secretion of labelled tracer occurred (**f**, arrowhead)

Bile leakage was detected by [^68^Ga]Ga-TEoS-DAZA PET/CT in 9 of 10 patients (90%) (Table 2). The identified leakage sites were variable and included central biliary structures such as the common hepatic duct (CHD, *n* = 1), right hepatic duct (RHD, *n* = 1), and BEA (*n* = 2, Fig. 1), as well as peripheral or postoperative sites such as the resection surface (*n* = 1), ducts of Luschka in the gallbladder bed (*n* = 2, Fig. 2), peripheral right hepatic ducts (*n* = 1, Fig. 3), and percutaneous fistula/drainage tracts (*n* = 1). In one patient (no. 5, Fig. 4), no bile leak was detected despite clinical suspicion. Leak detection after tracer administration was observed after a median of 21 min post injection (range 5–90 min). In one case, delayed detection at 90 min was associated with positional change. Bile leaks were classified according to the Nagano classification as type A (*n* = 1), type B (*n* = 4), and type C (*n* = 4). Cases not fully compatible with the original Nagano framework are indicated separately in Table 2.

**Table 2:** [^68^Ga]Ga-TEoS-DAZA PET/CT findings and subsequent treatment

Patient no.	Bile Leakage Site in [^68^Ga]Ga-TEoS-DAZA PET/CT	Leak detection time (min *p*.i.)	Nagano leak classification*	Subsequent treatment	Outcome measures
1	RHD	21	(C)	PTCD with stent placement	leak confirmed by PTC
2	CHD	55	C	ERCP with CHD stent placement	leak confirmed by selective ERC
3 (Fig. 1)	BEA	10	(C)	PTCD	decreasing external drainage output
4 (Fig. 2)	ducts of Luschka in gallbladder bed	25	(B)	BEA to bypass tumour-related CHD obstruction	leak confirmed by intraoperative fluoroscopy
5 (Fig. 4)	-	-	-	further external drainage	decreasing external drainage output
6	ducts of Luschka in gallbladder bed	90 (after patient repositioning)	(B)	ERCP with stent placement	clinical improvement
7	percutaneous fistula/drainage	12	(B)	PTCD	decreasing fistula output
8	resection surface	5	A	further external drainage	decreasing drainage output
9	BEA	27	(C)	further external drainage	decreasing drainage output
10 (Fig. 3)	peripheral right-hepatic bile ducts	13	B	ERCP with stent placement	leak confirmed by ERC

BEA: bilioenteric anastomosis; CHD: common hepatic duct; ERC(P): endoscopic retrograde cholangiopancreatography; PTC(D): percutaneous transhepatic cholangio(drainage); RHD: right hepatic duct. *Parentheses indicate non-standard Nagano classification cases

Physiological distribution of [^68^Ga]Ga-TEoS-DAZA showed tracer appearance in the intrahepatic bile ducts at 9.2 ± 3.5 (8.5, 3–16) minutes post injection, in the duodenum at 18.7 ± 12.0 (12.0, 7–44) minutes post injection, and renal excretion with activity in the renal pelvis at 6.6 ± 2.1 (6.0, 3–11) minutes post injection. In one patient, no tracer accumulation in the renal pelvis was observed within the first 20 min of imaging, in the context of markedly elevated creatinine levels and dialysis-dependent renal insufficiency.

PET/CT findings were considered to have informed subsequent clinical management in all patients. Management strategies included targeted interventions (ERCP with stent placement, PTCD with or without stent placement, surgical revision of BEA-related obstruction), or continuation or adjustment of external drainages (Table 2). Confirmation was obtained by invasive procedures in four cases (PTCD, ERCP, or intraoperative fluoroscopy), while five patients showed a decrease in external drainage or fistula output following the respective intervention. One additional patient demonstrated clinical improvement during follow-up.

## Discussion

This analysis aimed to explore the clinical feasibility of [^68^Ga]Ga-TEoS-DAZA PET/CT in patients with suspected bile leakage after hepatobiliary surgery. A detection rate of 90% in patients with prior negative or inconclusive imaging supports its clinical utility.

There is no standardised algorithm for the diagnosis of biliary leaks [1]. Ultrasound and CT are first-line imaging modalities for detecting intra-abdominal fluid collections; however, they cannot reliably differentiate bile leaks from other postoperative collections or accurately localise active leakage [2, 8]. ERCP and PTCD can detect bile leakages and enable simultaneous therapeutic interventions, but both are invasive procedures with a relevant risk of complications, including pancreatitis (particularly after ERCP), bleeding, and cholangitis (after PTCD) [9, 10]. In addition, they may fail to visualise ducts proximal or distal to an obstruction (e.g., stricture or stone). PTCD may also be technically challenging due to the frequent absence of intrahepatic duct dilatation [11]. MRCP is the non-invasive gold standard for morphological biliary imaging, providing detailed anatomical visualisation; when combined with hepatocyte-specific contrast-enhanced magnetic resonance imaging (MRI), it enables accurate functional assessment and localisation of bile leaks [2]. Nevertheless, limitations include suboptimal visualisation of peripheral intrahepatic ducts [12]. Interpretation may be confounded by technical and reconstruction artefacts (e.g., partial volume effects, respiratory motion) and by normal variants resembling pathology [13]. Moreover, MRCP is limited in patients with MRI-incompatible implants or devices and in those with claustrophobia [14]. In the immediate postoperative setting, image quality may additionally be compromised because recently operated patients are often unable to reliably perform the required breath-hold manoeuvres.

The spatial and temporal resolution of scintigraphic images is limited compared to PET/CT, which may require the use of higher-resolution imaging modalities [1, 15]. Hepatobiliary scintigraphy (HBS) is a known radionuclide diagnostic method for detecting bile leaks. The acquisition of sequential (or dynamic) images allows for tracking bile production in the liver and monitoring its flow through the biliary system into the small intestine. It can reveal the connection between the leak and any fluid collection as well as demonstrate the primary pathway of bile flow [2, 16]. Sensitivity of HBS has been reported to be lower in comparison to ERCP (50% and 79%, respectively), but at that time only planar scintigraphic images were compared [17]. At present, the use of single-photon emission computed tomography (SPECT/CT) should be a clinical standard and literature shows a significantly higher diagnostic accuracy for SPECT/CT compared to planar scintigraphic imaging with rates of 96.8% versus 65.6%, and no false-positive results reported for SPECT/CT [18]. Theoretically, HBS should be useful to determine the presence or absence of a leak, but the relatively fast hepatic metabolism of mebrofenin combined with the long acquisition times of SPECT and the low resolution often make it difficult to pinpoint the exact source of a leak regarding overlapping anatomical structures [19].

The novel PET tracer [^68^Ga]Ga-TEoS-DAZA can be synthesised according to GMP guidelines and has been applied for hepatobiliary imaging in the evaluation of bile duct patency and localisation of a traumatic biliary leak [20–22]. Administration of 150 MBq (4.05 mCi) of gallium-68 to a 75-kg adult results in an estimated whole-body effective radiation dose of approximately 3.15 mSv. The low-dose abdominal CT scan performed for attenuation correction contributes an additional effective dose of approximately 0.8-2 mSv [23]. Not all patients required the full 60-minute imaging protocol. As the PET protocol consisted of consecutive 20-minute acquisitions, each completed segment could be reconstructed and reviewed while the subsequent acquisition was still ongoing. This enabled near real-time assessment of the images, preferably in joint review with the surgical team, allowing the examination to be terminated early once sufficient diagnostic information had been obtained. Conversely, if no bile leak was identified after 60 min, additional 20-minute acquisitions could be performed. In selected cases, brief patient mobilisation before further imaging may facilitate leak detection.

A recent analysis of five patients after liver transplantation demonstrated the utility of dynamic [^68^Ga]Ga-TEoS-DAZA PET/CT for bile leak localisation [24]. In our cohort, the location of bile leakage was successfully identified in 9 of 10 patients. The detected bile leak locations were diverse, underscoring the method’s ability to depict even complex or atypical leaks. Moreover, it may have the potential to overcome diagnostic limitations by visualising bile ducts that are not readily accessible by endoscopy, particularly peripheral leaks. Unlike ERCP, which relies on retrograde contrast injection, [^68^Ga]Ga-TEoS-DAZA PET/CT provides antegrade depiction of the biliary system along the physiological bile flow. This approach may provide a more comprehensive assessment of biliary anatomy and function. Notably, [^68^Ga]Ga-TEoS-DAZA PET/CT successfully identified bile leaks from Luschka (subvesicular) ducts, a frequently overlooked and diagnostically challenging source of postoperative bile leakage [25]. In one case, delayed detection at 90 min was possible after positional change (intermittent standing), suggesting dynamic or low-flow leakage. In the patient without detectable bile leak (patient 5), a complex history (including recurrent hepatic cysts, common bile duct (CBD) laceration during fenestration, and subsequent BEA formation) was noted. Right hepatic lobe atrophy with cystic changes and chronic occlusion of the right hepatic artery and portal vein likely led to reduced tracer uptake and excretion. Moreover, the leak volume at the time of [^68^Ga]Ga-TEoS-DAZA PET/CT was presumably too low for detection, representing a limitation of the method. In our experience, the method depends on adequate biliary excretory function of the liver. In the presence of elevated bilirubin levels, the radiotracer competes with bilirubin for hepatocellular uptake via the OATP1B3 transporter [5]. Hepatocyte transporter studies showed significant [^68^Ga]Ga-TEoS-DAZA uptake in cells expressing OATP1B3. Moreover, ex vivo biodistribution studies in ostrich embryos demonstrated a notable reduction in hepatic uptake of [^68^Ga]Ga-TEoS-DAZA following co-administration of cyclosporine A, which may be attributable to OATP1B3 inhibition. However, a threshold for inhibition related to elevated serum bilirubin levels has not yet been established and is currently based only on clinical observations. Notably, the laboratory liver function of patient 5 was normal (e.g. bilirubin level 12 µmol/L) because the impaired function of the right liver lobe was still compensated for by the hypertrophic left liver lobe. Further investigations are ongoing to better understand the relationship between [^68^Ga]Ga-TEoS-DAZA uptake in hepatocytes and different aspects of the liver function.

Importantly, [^68^Ga]Ga-TEoS-DAZA PET/CT contributed to clinical decision-making and included management strategies guided by the localisation of the identified leak sites, whereas in the patient without PET evidence of a bile leak, the examination also supported continued drainage management without immediate escalation to further invasive procedures.

Limitations of this analysis include the small sample size and its single-centre design, warranting validation in larger cohorts. Since [68Ga]Ga-TEoS-DAZA PET/CT were performed in a clinical setting, no systematic comparison with established hepatobiliary imaging modalities, including HBS and hepatobiliary contrast-enhanced MRI, was possible. Future studies should therefore include prospective comparative evaluations of these techniques. A formal interobserver analysis should be incorporated into subsequent investigations to assess reproducibility and reader agreement. PET/CT availability remains limited to specialised centres, and radiation exposure should be acknowledged, although it is of minor concern in this clinical setting. While no false positives were observed, aberrant bile ducts may be misinterpreted as leaks in complex postoperative cases. No extra-hepatobiliary or non-renal tracer accumulation has been observed to date.

Based on our current experience, [^68^Ga]Ga-TEoS-DAZA PET/CT is not intended to replace established imaging modalities but may serve as a problem-solving tool in selected patients with persistent clinical suspicion of bile leakage despite inconclusive conventional imaging. Its role may be particularly relevant in complex postoperative settings, altered anatomy precluding successful cholangiography, or when the site of leakage remains uncertain despite prior imaging.

## Conclusion

Cholangio-PET/CT with [^68^Ga]Ga-TEoS-DAZA demonstrated high clinical yield for the detection and localisation of postoperative bile leaks in this selected cohort, including complex and peripheral leak sites. Its ability to provide dynamic, antegrade functional imaging of the biliary system may offer clinically useful information, particularly in patients with inconclusive conventional imaging findings, and directly impacts clinical decision-making. Despite limitations related to sample size and availability, these findings support the potential of [^68^Ga]Ga-TEoS-DAZA PET/CT as a valuable tool in the diagnostic workup of suspected bile leaks, warranting further validation in larger, comparative studies.

## Data Availability

The datasets analysed during the current study are available from the corresponding author on reasonable request.
